# Muscle damage and inflammation after eccentric exercise: can the repeated bout effect be removed?

**DOI:** 10.14814/phy2.12648

**Published:** 2015-12-10

**Authors:** Nikos V. Margaritelis, Anastasios A. Theodorou, Vasilios Baltzopoulos, Constantinos N. Maganaris, Vassilis Paschalis, Antonios Kyparos, Michalis G. Nikolaidis

**Affiliations:** ^1^ School of Physical Education and Sports Science at SerresAristotle University of ThessalonikiSerresGreece; ^2^ Department of Health SciencesEuropean University CyprusNicosiaCyprus; ^3^ Department of Life SciencesBrunel University LondonUxbridgeUK; ^4^ Research Institute for Sport and Exercise SciencesLiverpool John Moores UniversityLiverpoolUK; ^5^ School of Physical Education and Sport ScienceUniversity of ThessalyTrikalaGreece

**Keywords:** Adaptation, eccentric exercise, repeated bout effect

## Abstract

The current consensus in exercise physiology is that the repeated bout effect always appears after few eccentric exercise sessions. This is the first attempt to challenge this tenet, by exploiting specificity in muscle plasticity. More specifically, we examined whether the opposing adaptations in muscle induced after concentric and eccentric exercise can attenuate and/or remove the repeated bout effect. Seventeen young men were randomly assigned into one of the following groups: (1) the alternating eccentric‐concentric exercise group; and (2) the eccentric‐only exercise group. Both groups performed 8 weeks of resistance exercise using the knee extensors of both legs on an isokinetic dynamometer. The alternating eccentric‐concentric exercise group performed an alternating exercise protocol, switching between eccentric‐only and concentric‐only exercise every 4 weeks, while the eccentric‐only group performed eccentric exercise. Evaluation of muscle damage using physiological (isometric torque, delayed onset muscle soreness, and range of movement) and biochemical (creatine kinase) markers and inflammation (C‐reactive protein) was performed at weeks 1, 5, and 10. Baseline isometric peak torque was also evaluated at week 14 after another cycle (4 weeks) of alternating or eccentric‐only exercise training. In the alternating eccentric‐concentric exercise group, the concentric exercise training performed prior to eccentric exercise reduced dramatically the repeated bout effect by reversing muscle back to its unaccustomed state. On the contrary, the eccentric‐only exercise group exhibited a typical manifestation of the repeated bout effect. Interestingly, muscle strength was elevated similarly for both alternating and eccentric‐only exercise groups after 13 weeks of training. The alternating eccentric‐concentric exercise scheme, implemented in the present study, has for the first time successfully overcame the repeated bout effect. The similarity in muscle strength measurements following the two protocols is against the notion that inflammation plays an important role in exercise‐induced adaptations in muscle.

## Introduction

Eccentric exercise is linked to several health‐promoting adaptations. For example, chronic eccentric exercise elicits many beneficial effects, such as favorable changes in blood lipid profile (Nikolaidis et al. 2008; Panayiotou et al. 2013), improved oxidative stress status (Theodorou et al. 2011), and improved insulin sensitivity (Paschalis et al. 2011). Eccentric exercise also has beneficial effects on inflammatory status. Although the eccentric exercise‐induced muscle microdamage acutely upregulates pro‐inflammatory/pro‐oxidant agents (e.g., increased IL‐6), chronically over a longer term, it down‐regulates pro‐inflammatory/pro‐oxidant agents and upregulates anti‐inflammatory/anti‐oxidant agents (e.g., increased IL‐10; [Paulsen et al. 2012]). It has been suggested that these beneficial effects of eccentric exercise are partially mediated by the muscle fiber microdamage induced by the stretch experienced during the application of the higher forces generated during eccentric actions (Nikolaidis et al. 2008; Paschalis et al. 2011; Theodorou et al. 2011; Panayiotou et al. 2013). It was further suggested that the muscle damage induced by eccentric exercise also causes greater increases in muscle strength compared to concentric exercise due to the greater muscle hypertrophy that follows eccentric training compared to concentric (Hortobagyi et al. 1996; Farthing and Chilibeck 2003). This stretch induced microdamage appears, therefore, to be a main mechanism or mechanical trigger for the beneficial adaptations of eccentric exercise. However, after an initial period of rapid favorable adaptations in health related parameters, the beneficial impact of eccentric exercise is lost or diminished with subsequent eccentric exercise sessions. This loss of beneficial adaptations is linked to the reduction in stretch induced damage, with minimal or no muscle damage after only eight repeated sessions of eccentric exercise (Nikolaidis et al. 2008; Mackey et al. 2011; Paschalis et al. 2011; Theodorou et al. 2011; Panayiotou et al. 2013).

This phenomenon of reduced damage with repeated eccentric exercise sessions is the so‐called “repeated bout effect”, which represents a physiological “absolute norm” associated with eccentric exercise. Another remarkable characteristic of the repeated bout effect is that its protective effects last up to 6 months after the first bout, even without any exercise in between (Nosaka et al. 2001). The appearance of this effect after few unaccustomed eccentric exercise sessions has now been established as one of the key tenets of exercise physiology and a mechanism for muscle damage protection (Mackey et al. 2011; Hyldahl et al. 2015). However, this protective effect is also a barrier for using eccentric exercise as a continuous stimulus for the beneficial and distinct long‐term adaptations associated with muscle‐damage induced pathways as explained above.

The main question is whether the manifestation of the repeated bout effect is an innate biological protection mechanism or just the consequence of the type of muscle contraction used. So, we wanted to examine if the repeated bout effect is actually the result of the training modality used to study this phenomenon (repeated eccentric cycles only) or if muscle plasticity can be exploited for removing the repeated bout effect by interjecting concentric exercise cycles between the eccentric bouts. This idea is based on indications of previous studies showing that a prior period of concentric exercise renders the muscle more vulnerable to damage with subsequent eccentric loading (Ploutz‐Snyder et al. 1998; Whitehead et al. 1998; Gleeson et al. 2003). Our proposal is based on the premise that considers muscle microdamage as the mechanical trigger for positive biochemical and physiological health adaptations that needs to be preserved with repeated eccentric exercise sessions rather than prevented or reduced through the protection provided by the repeated bout effect.

In order to test this principle, that can form the basis for long‐term eccentric exercise beneficial effects, we designed an exercise protocol in which the “muscle damaging” eccentric‐only exercise was followed by an equally lasting period of concentric‐only, that is “nonmuscle damaging” exercise. We hypothesized that this alternating contraction scheme (eccentric‐concentric‐eccentric) would enable the second eccentric exercise bout to acquire again its “unaccustomed” character and lead to muscle microdamage, thus attenuating and/or removing the repeated bout effect.

## Methods

### Ethical approval

An informed written consent was obtained for all participants, after they were informed of all risks, discomforts and benefits involved in the study. The procedures were in accordance with the 1975 Declaration of Helsinki, as revised in 2000, and ethical approval was received from the Ethics Committee of the European University Cyprus (#018/27‐3‐2014).

### Participants

Seventeen (*N* = 17) young men participated in the present investigation. Individuals were randomly assigned into one of the following two groups: (1) the alternating eccentric‐concentric exercise group (*n* = 9, age 22 ± 3 years, height 177 ± 5 cm, mass 76 ± 7 kg; mean ± SD); and (2) the eccentric‐only exercise group (*n* = 8, age 23 ± 2 years, height 176 ± 7 cm, mass 74 ± 6 kg; mean ± SD). During the exercise sessions or the days after the exercise, participants were asked to immediately report any serious adverse events to the investigator. Subjects were excluded from the study, if they had any muscle disease or a prior history of musculoskeletal injury to the lower limbs that would limit the ability to perform the exercise sessions.

Participants were moderately active, had a sedentary job and participated in low intensity leisure activities (such as swimming and dancing) two to three times per week for less than three hours per week. Subjects had no experience with muscle damaging exercise for at least 6 months before the study and were not taking any anti‐inflammatory or analgesic medications or nutritional supplements during the study period and 1 month before the initiation of the experiment. Volunteers were also instructed to abstain from any strenuous exercise (except for the exercise sessions performed during the experimental intervention) during their participation in the study.

### Study design

**Figure 1:**
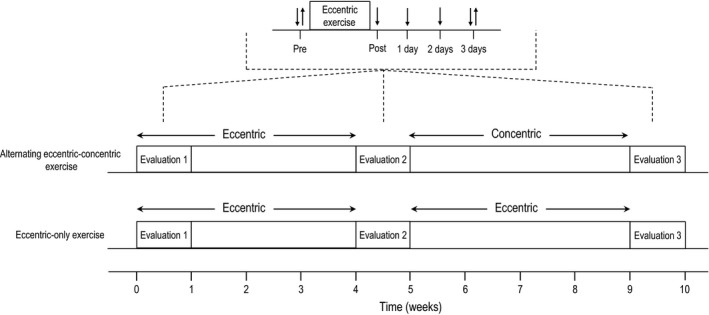
Study design. Downward arrows indicate the time of physiological measurements and upward arrows indicate the time of biochemical measurements.

The two experimental groups performed 8 weeks of resistance exercise using the knee extensors of both legs (Fig. 1). The alternating eccentric‐concentric exercise group performed an alternating exercise protocol, switching between eccentric‐only and concentric‐only exercise sessions every 4 weeks on an isokinetic dynamometer (Cybex Norm, NY). The eccentric‐only group performed eccentric muscle actions using the same isokinetic dynamometer. The eccentric exercise sessions for both groups were performed only once a week, whereas the concentric exercise sessions in the alternating exercise protocol were performed three times per week. Concentric training was performed three times per week, based on the position stand published by the American College of Sports Medicine (2009), which recommends this frequency of resistance exercise for improving muscle strength in previously untrained individuals (as was the case in the present study). On the other hand, eccentric training was performed once per week based on a protocol already used in previous similar studies (Paschalis et al. 2011; Theodorou et al. 2011). In particular, it was observed that when eccentric exercise was performed more than once per week, muscle function was not fully recovered due to muscle damage (Paschalis et al. 2011; Theodorou et al. 2011).

Physiological markers of muscle function and muscle damage (isometric torque, DOMS and range of movement; ROM) were measured pre‐exercise, immediately post eccentric exercise, 1, 2, and 3 days postexercise. Biochemical markers of muscle damage (creatine kinase; CK) and inflammation (C‐reactive protein; CRP) were measured pre‐exercise and 3 days postexercise, due to the fact that both CK (Nikolaidis et al. 2007; Paschalis et al. 2011) and CRP (Brull et al. 2003) peak at about 3 days after an eccentric exercise session. Evaluation of muscle damage and inflammation was performed at week 1, week 5, and week 10. For a comprehensive evaluation of muscle strength after the alternating and eccentric‐only exercise protocols, baseline isometric peak torque was performed at week 14 after another cycle (4 weeks) of alternating or eccentric‐only exercise training. There is no doubt that mechanistic/structural measurements could have increased the reliability of muscle damage assessment. However, in a relevant review by Warren et al. (1999) it was concluded that measurement of peak torque and range of movement (which both measured in the present study) are two of the most reliable methods for quantifying muscle injury.

### Exercise protocols

The exercise sessions were performed on an isokinetic dynamometer (Cybex Norm, Ronkonkoma, NY). The exercise protocols were undertaken from the seated position (120° hip angle), after the participants were stabilized according to the manufacturer's instructions. During sessions, participants had to complete 5 sets of 14 contractions (70 contractions in total) with the knee extensors of both legs at an angular velocity of 60°/s (knee range, 0° [full extension] to 90° flexion). A 2‐min rest interval was utilized between sets. Both the alternating and the eccentric‐only exercise protocols consisted of maximal voluntary contractions.

### Measurements

The isokinetic dynamometer was used for the measurement of isometric knee extensor peak torque at 90° knee flexion. The average of the three best maximal voluntary contractions with the preferred leg was recorded. To ensure that the subjects provided their maximal effort, the measurements were repeated if the difference between the lower and the higher torque values exceeded 10%. There was a 2‐min rest between isometric efforts. The test–retest reliability of the isometric peak torque measurement was 0.98. The assessment of pain‐free ROM was performed manually. The investigator moved the calf at a very low angular velocity from 0° knee extension to the position where the subject felt any discomfort. The test–retest reliability of the ROM measurement was 0.93. Each participant assessed DOMS during a squat movement (90° knee flexion), and perceived soreness was rated on a visual analog scale ranging from 1 (normal) to 10 (very sore) (Nikolaidis et al. 2008; Paschalis et al. 2011). The test‐retest reliability of the DOMS measurement was 0.94. Blood sample was drawn from a forearm vein and collected in EDTA tubes. Blood was immediately centrifuged at 1370 g for 10 min at 4°C and plasma was collected for the assessment of CK and CRP. Samples were stored at −80°C and thawed only once before analysis. Creatine kinase was assessed in plasma in a COMBAS Integra Plus 400 chemistry analyzer (RocheDiagnostics, Mannheim, Germany). Due to the limitation of COMBAS analyzers to provide accurate values of CK activity above 500 U/l, all post exercise samples were diluted 10 times. C‐reactive protein was assessed using an ELISA kit (RAB0096‐1KT, Sigma‐Aldrich Co., St. Louis).

### Data analysis

The distribution of all dependent variables was examined by Shapiro‐Wilk test and was found not to differ significantly from normality. Differences on physical characteristics between the groups at baseline were examined by using an unpaired Student's *t* test. Two‐way ANOVA (group × time) with repeated measurements on time were used to examine the effect of both training protocols on muscle strength (baseline assessment at the 1st and at the 14th week). Three separate two‐way ANOVAs (group × time) with repeated measurements on time were used to examine the effect of alternating or eccentric‐only exercise on CK and CRP (before and at day 3 postexercise), as well as on isometric torque, ROM and DOMS (before, immediately after and at days 1, 2 and 3 post eccentric exercise) at weeks 1, 5, and 10. If a significant interaction was obtained, pairwise comparisons were performed through simple main effect analysis. Epsilon value of Mauchly's sphericity test was above 0.73 for all ANOVA analyses. The statistical power required for detecting a 20% post‐exercise change in each biomarker was calculated using G*Power 3 (Faul et al. 2007). The pooled standard deviation of the resting and exercise values was used for these calculations, while the *α* level was set at 0.05. Data are presented as mean±SEM. The SPSS version 18.0 was used for all analyses (SPSS Inc., Chicago, Illinois).

## Results

### Physical characteristics

No differences in physical characteristics at baseline between the two groups were observed (data not shown).

### Muscle damage and inflammation

**Figure 2:**
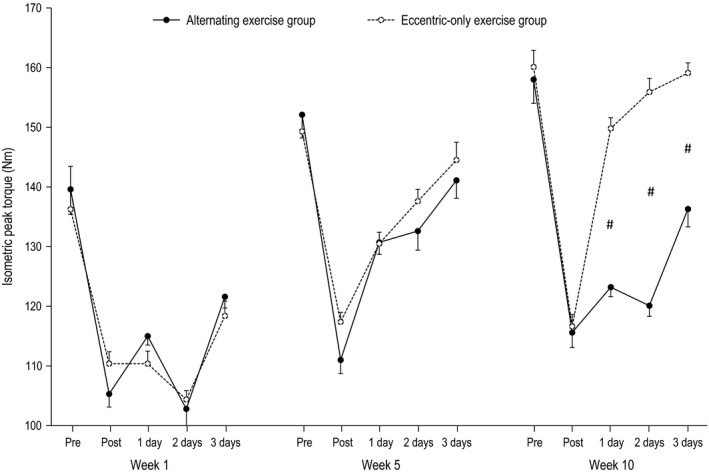
Isometric torque in the alternating eccentric‐concentric exercise group (closed circles) and eccentric‐only exercise group (open circles) (mean ± SEM). #Indicates significant difference at the same time point between the two groups.

**Figure 3:**
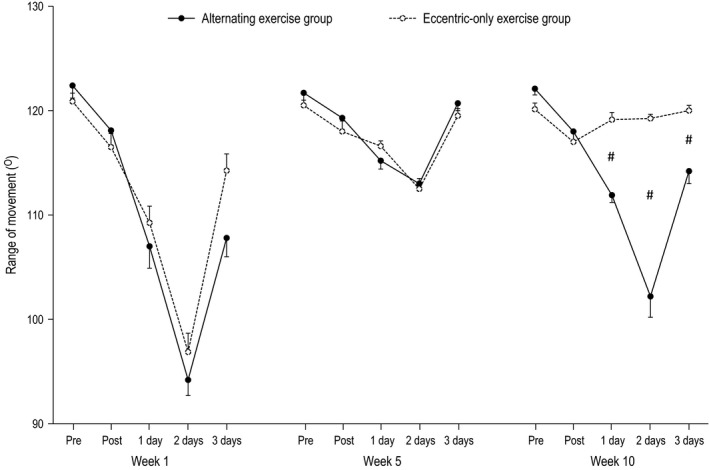
Range of movement in the alternating eccentric‐concentric exercise group (closed circles) and eccentric‐only exercise group (open circles) (mean ± SEM). #Indicates significant difference at the same time point between the two groups.

**Figure 4:**
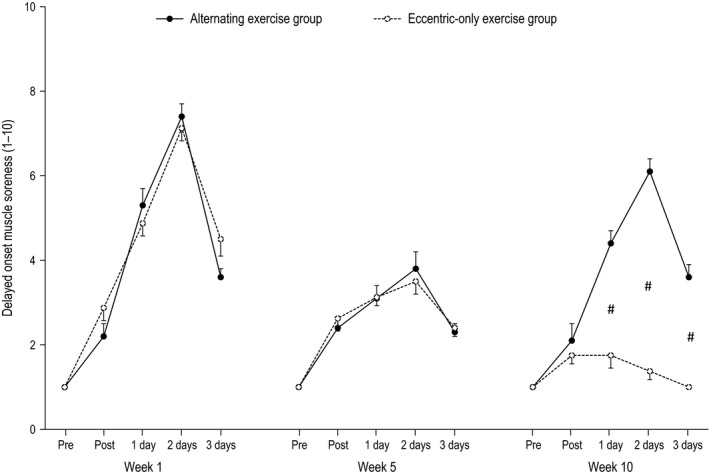
Delayed onset muscle soreness in the alternating eccentric‐concentric exercise group (closed circles) and eccentric‐only exercise group (open circles) (mean ± SEM). #Indicates significant difference at the same time point between the two groups.

**Figure 5:**
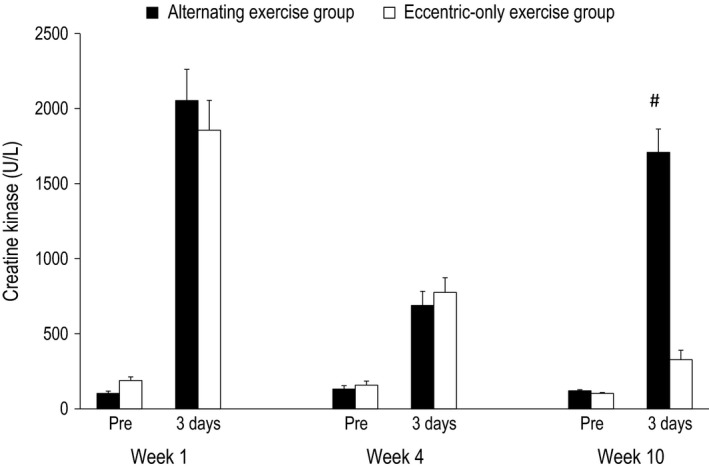
Creatine kinase in the alternating eccentric‐concentric exercise group (black bars) and eccentric‐only exercise group (white bars) (mean ± SEM). #Indicates significant difference at the same time point between the two groups.

**Figure 6:**
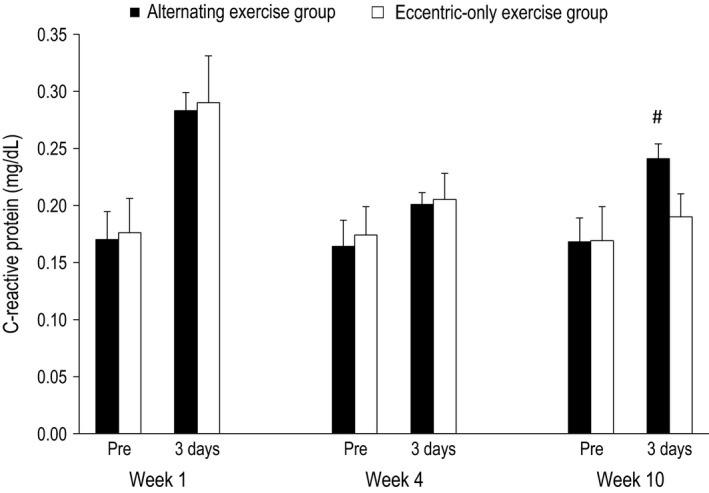
C‐reactive protein in the alternating eccentric‐concentric exercise group (black bars) and eccentric‐only exercise group (white bars) (mean ± SEM). #Indicates significant difference at the same time point between the two groups.

The effect of alternating eccentric‐concentric exercise and eccentric‐only exercise on isometric torque, ROM, DOMS, CK, and CRP are presented in Figures 2, 3, 4, 5, 6. The statistical power required to detect a 20% postexercise change after the first eccentric exercise bout in the alternating exercise group was 97% for isometric peak torque, 95% for ROM, 100% for DOMS and 98% for CK. The respective values for the eccentric‐only exercise group were 98% for torque, 100% for ROM, 100% for DOMS and 93% for CK. The statistical power after the first eccentric exercise bout in the alternating exercise group was 97% for isometric peak torque, 95% for ROM, 100% for DOMS and 98% for CK. The respective values for the eccentric‐only exercise group were 98% for torque, 100% for ROM, 100% for DOMS and 93% for CK.

#### Week 1

For all dependent variables, a main effect of time (*P* < 0.001) appeared after exercise. In particular, all muscle damage indices altered post exercise compared to the pre‐exercise values in both the alternating (i.e., isometric torque and ROM decreased by −26% and −23%, respectively, while DOMS and CK increased by 640% and 1889%, respectively) and the eccentric‐only exercise group (i.e., isometric torque and ROM decreased by −23% and −20%, respectively, while DOMS and CK increased by 610% and 880% respectively). The inflammatory marker CRP was increased by 67% and 65% for the alternating and the eccentric‐only exercise group, respectively. No main effect of group nor a significant interaction group × time was observed.

#### Week 5

Likewise to week 1, a main effect of time (*P* < 0.001 except for CRP where *P* = 0.004) appeared after exercise for all dependent variables. Muscle damage indices altered postexercise compared to the pre‐exercise values in both the alternating (i.e., isometric torque and ROM decreased by −14% and −7%, respectively, while DOMS and CK increased by 280% and 422%, respectively) and the eccentric‐only exercise group (i.e., isometric torque and ROM decreased by −13% and −6%, respectively, while DOMS and CK increased by 250% and 389% respectively). The inflammatory marker CRP was increased by 23% and 18% for the alternating and the eccentric‐only exercise group respectively. As in week 1, no main effect of group nor a significant interaction group × time was observed.

#### Week 10

A main effect of time was observed for all dependent variables (*P* < 0.001), while a main effect of group appeared for DOMS, CK and ROM (*P* < 0.001) as well as for isometric torque (*P* = 0.005). However, no main effect of group was observed for CRP (*P* = 0.392). Moreover, a significant group × time interaction was observed for all dependent variables (*P* < 0.001 except for CRP where *P* = 0.022). In detail, muscle damage indices markedly changed postexercise compared to the pre‐exercise values in the alternating group (i.e., isometric torque and ROM decreased by −24%, −16%, respectively, while DOMS and CK increased by 510% and 1316% respectively). On the contrary, the corresponding changes were minor in the eccentric‐only exercise group (i.e., isometric torque and ROM decreased by −6%, −1%, respectively, while DOMS and CK increased by 80% and 215% respectively). Following the same pattern, the inflammatory marker CRP increased by 44% in the alternating exercise group while the increase was only 12% in the eccentric‐only exercise group.

#### Week 14

**Figure 7:**
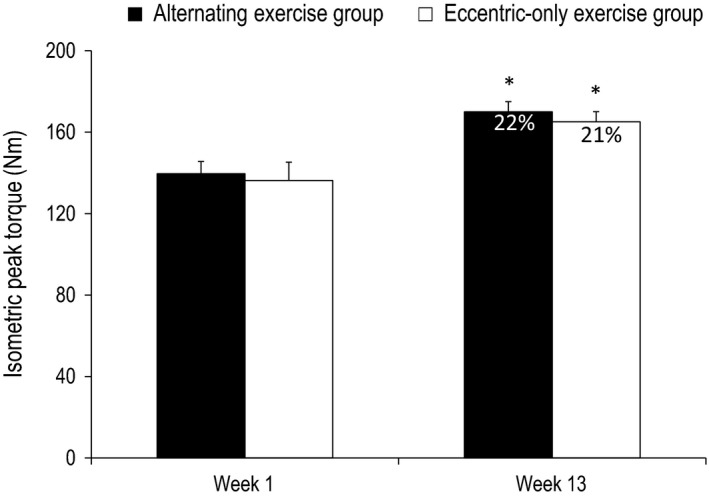
Baseline assessment of isometric torque in the alternating exercise group (black bars) and eccentric‐only exercise group (white bars) at the 1st and at the 14th week (mean ± SEM). Percent values indicate the relative change of isometric torque compared to baseline values. *Indicates significant difference compared to baseline values.

At week 14, after another cycle of alternating or eccentric‐only exercise (i.e., from 10th to 13th week of training), the baseline peak torque was found significantly increased in the alternating exercise group compared to the initial values by 22% (139 Nm vs. 170 Nm; *P* < 0.001). Similarly, the baseline peak torque was also found significantly increased in the eccentric‐only exercise group compared to the initial values by 21% (136 Nm vs. 165 Nm; *P* = 0.006). As a result, no difference was observed between the groups in peak torque after 13 weeks of training (*P* = 0.259) (Fig. 7), indicating that both alternating and eccentric‐only training had similar impact on muscle strength improvement.

## Discussion

The current consensus in exercise physiology is that the repeated bout effect inevitably appears after few eccentric exercise sessions. This is the first attempt to challenge this tenet, by attributing this phenomenon to the specificity in muscle plasticity. More specifically, we examined whether the opposing muscle adaptations induced by either concentric or eccentric exercise can attenuate or even totally remove the repeated bout effect. The main finding of this study is that an equal lasting period of concentric exercise prior to eccentric exercise reduces dramatically the repeated bout effect. This novel alternating exercise protocol seems to reverse muscle back to its unaccustomed state within only a month, contrary to the widely accepted notion of the long‐lasting effects of the repeated bout effect. Interestingly, muscle strength was increased similarly after the alternating and the eccentric‐only exercise despite the fact that the alternating exercise group experienced repeated cycles of muscle damage.

### Attenuating/removing the repeated bout effect

Confirming our hypothesis, the muscle‐damage markers in the two experimental groups followed different patterns after exercise. The eccentric‐only exercise group exhibited a typical manifestation of the repeated bout effect, namely a gradual reduction of muscle damage after repeated eccentric sessions. More specifically, all markers of muscle damage at week 5, although significantly altered, showed considerably smaller changes compared to these of the initial eccentric exercise session (week 1). Moreover, at week 10, all markers of muscle damage (except for CK) showed minimal non‐significant changes after exercise. This indicates that eccentric exercise lost its unaccustomed “muscle damaging” character completely after 10 eccentric exercise sessions. These data are in line with previous findings from our group showing absence of muscle damage after chronic eccentric exercise of similar duration (Paschalis et al. 2011; Theodorou et al. 2011). Similarly, the group performing the alternating eccentric‐concentric exercise scheme showed a gradual reduction in muscle damage during the first 5 weeks (indicating the appearance of the repeated bout effect). However, and in contrast with the eccentric‐only group, the subsequent 4 weeks of concentric‐only exercise was sufficient to bring muscle nearly back to its unaccustomed state. Indeed, the last eccentric exercise session (week 10), which was preceded by a month of concentric‐only exercise, induced muscle damage almost to the same level as that experienced after the first eccentric exercise session at week 1. These findings reasonably indicate an almost complete removal of the repeated bout effect.

The alternating eccentric‐concentric exercise scheme caused a repeated episode of muscle damage and, as a consequence, it also induced a repeated episode of inflammation (measured by CRP). As it was expected, after the fifth session of eccentric exercise, the increase of CRP levels was much lower compared to the first eccentric exercise session indicating the appearance of the repeated bout effect. As was the case with muscle damage, one month of concentric exercise was enough to bring muscle nearly back to an unaccustomed state, as signified by the 44% increase in CRP levels after the last eccentric exercise session (week 10). For comparison, in the eccentric‐only group after the last eccentric exercise session the respective change in CRP levels was negligible (i.e., approximately 4‐fold lower compared to the alternating group), revealing minimal inflammation. Although seemingly detrimental, the current scientific consensus suggests that the acute inflammatory episodes after exercise are transient and become the signal for the organism to respond by producing anti‐inflammatory and other beneficial adaptations (Evans and Cannon 1991; Ward et al. 1999; Raschke and Eckel 2013; Trappe and Liu 2013). This favorable effect of inflammation may be explained by the “stress‐response” hormesis theory, which asserts that brief exposure to a stressor can be beneficial through induction of adaptive mechanisms increasing the tolerance against future stress incidents (Gems and Partridge 2008). However, considering that the continuous addition and removal of sarcomeres is energetically highly expensive and the chronic systemic inflammatory environment might lead to maladaptations (Fry and Kraemer 1997), we cannot ascertain that the repeated episodes of muscle damage and inflammation will be ultimately beneficial, especially if the alternating eccentric‐concentric exercise scheme is performed for a long time period.

### Mechanisms of attenuation/removal of the repeated bout effect

**Figure 8:**
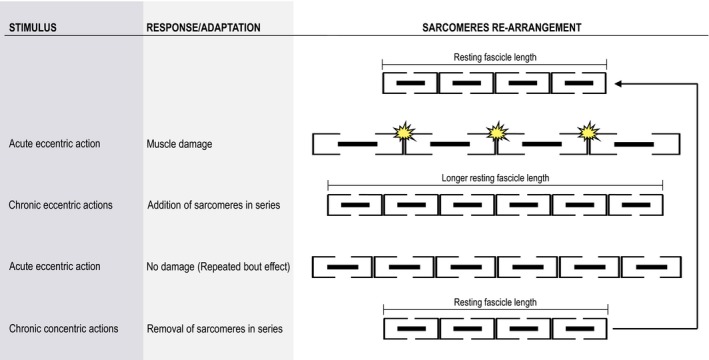
A conceptual model, based on our and others’ experimental findings, showing how alternating eccentric‐concentric exercise can remove or reduce the repeated bout effect. It is well‐known that skeletal muscle adapts to a new functional length by changing the number of sarcomeres in series (Williams and Goldspink 1971; Tabary et al. 1972). As a result, after an initial period of eccentric exercise‐induced muscle damage, skeletal muscle adapts to chronic eccentric exercise by adding sarcomeres in series restoring fascicle length (i.e., longer resting fascicle length; [Butterfield and Herzog 2006; Reeves et al. 2009]). This leads to absence of muscle damage after subsequent eccentric exercise, signifying the appearance of the repeated bout effect. On the contrary, the following concentric‐only exercise period results in loss of sarcomeres in series (Butterfield and Herzog 2006), which shifts the length‐tension relationship in the direction of shorter lengths, increasing muscle stiffness (Lynn and Morgan 1994; Talbot and Morgan 1998; Whitehead et al. 1998; Timmins et al. 2015). Therefore, any subsequent eccentric contraction place the fewer serial sarcomeres into a length range in which they are overstretched and therefore more vulnerable to damage (Talbot and Morgan 1998; Whitehead et al. 1998; Lau et al. 2015). Conclusively, the alternating eccentric‐concentric exercise model exploits the finding that concentric only exercise renders the muscle more vulnerable to damage after any subsequent eccentric exercise (Ploutz‐Snyder et al. 1998; Whitehead et al. 1998; Gleeson et al. 2003; Butterfield and Herzog 2006).

What molecular re‐arrangements at the sarcomere level may have caused the removal of the repeated bout effect? Based on relevant experimental evidence, a tempting hypothesis is that chronic eccentric exercise leads to sarcomerogenesis (i.e, addition of serial sarcomeres) leading to less stretch during an eccentric action, whereas the following chronic concentric exercise induces sarcomerolysis (i.e, removal of serial sarcomeres) leading to more stretch during the subsequent eccentric action (Williams and Goldspink 1971; Tabary et al. 1972; Ploutz‐Snyder et al. 1998; Whitehead et al. 1998; Gleeson et al. 2003; Butterfield et al. 2005; Timmins et al. 2015; Lau et al. 2015). The successive application of concentric exercise followed by eccentric exercise renders the sarcomeres more vulnerable to stretch‐induced damage in the subsequent eccentric exercise overcoming the repeated bout effect (Fig. 8). A corollary to this hypothesis is that the repeated bout effect may be simply the outcome of the training modality. Consequently, switching between equal periods of eccentric‐only and concentric‐only exercise leads to different adaptations and strains at the muscle fascicle and sarcomere levels.

Experimental measurements of sarcomere operating length in humans are invasive and impractical (Lieber and Friden 1998). However, indirect measures of muscle fiber length can be taken in vivo and a recent study has shown that the mechanism underpinning the repeated bout effect is the reduced magnitude of muscle fascicle lengthening during a repeated eccentric exercise bout compared to the first eccentric session (Lau et al. 2015). Other studies have shown that eccentric and concentric exercise training modes induce divergent adaptations in human muscle architecture. Specifically, in a 10‐week training study of the quadriceps muscle, it has been shown that eccentric loading only produces larger increases in fascicle length but smaller increases in pennation angle than concentric loading, despite the two training modes producing similar increases in muscle mass (Franchi et al. 2014). In an earlier study, Blazevich et al. (Blazevich et al. 2007) showed that 10 weeks of eccentric‐only or concentric‐only training of the knee extensors resulted in similar increases in fascicle length and pennation angle. Notwithstanding the smaller increase in fascicular length with concentric training in the study of Franchi et al. (Franchi et al. 2014), the qualitative interpretation and mechanism underpinning the macroscopic adaptation of a longer muscle fascicle would be that new sarcomeres were added in‐series, which stands in contrast with the findings of all the studies above which clearly show that short‐term concentric training results in sarcomerolysis. However, it must be noted that the concentric training in these studies lasted for only 5 days, while in our study and the study of Franchi et al. (2014) and Blazevich et al. (2007) the duration was much longer, 4 and 10 weeks respectively. It is unlikely that 5 days of concentric training were sufficient to produce hypertrophy, but the longer training duration studied here may be effective in producing a measurable increase in muscle mass. Nevertheless, it is reasonable to suggest that, the average sarcomere in the present study would have operated at a longer and more vulnerable length during eccentric exercise following the concentric training implemented, as evidenced by the extensive muscle damage present.

### Muscle damage and improvements in muscle strength

The initial eccentric exercise session induced “muscle microdamage” and caused disturbances in muscle performance in both groups. The increase in muscle strength, measured as average torque output, after the 13 weeks of alternating (repeated episodes of “muscle microdamage”) or eccentric‐only (repeated bout effect) training was similar for both training groups (22% and 21% respectively). The comparable increase in muscle strength observed between the two training groups is against the notion of muscle damage as being the predominant stimulus for muscle strength (Hortobagyi et al. 1996; Farthing and Chilibeck 2003; Hilliard‐Robertson et al. 2003) and shows that muscle strength can occur independently of any symptoms of muscle damage (Blazevich et al. 2007; Nickols‐Richardson et al. 2007; Flann et al. 2011). It is worth noting, however, that the alternating group had a larger volume of training because of the intervening concentric training period. One of the reasons for the similarity in strength improvement between the two groups could be the inability of the participants in the alternating group to train with maximal intensity due to the increased muscle microdamage induced in this group.

## Conclusion

Unaccustomed eccentric exercise is known to cause widespread muscle damage. An “absolute norm” in eccentric exercise is the so‐called “repeated bout effect”, which manifests as a gradual reduction of muscle damage after repeated eccentric exercise sessions. The alternating eccentric‐concentric exercise scheme that we devised and implemented in the present study, questioned this “dogma” for the first time by almost completely removing this adaptive phenomenon and perpetuating repeated episodes of muscle damage and inflammation with repeated eccentric cycles. This was accomplished by exploiting muscle mechanics specific adaptations during concentric‐only and eccentric‐only exercise. This novel paradigm has successfully overcame the repeated bout effect, making experimentally feasible to investigate the potential role of exercise‐induced muscle microdamage as a stimulus for physiological and biochemical adaptations. Fewer eccentric exercise sessions preceded by concentric exercise sessions induced similar improvements in muscle strength with a higher number of eccentric exercise sessions, indicating that muscle damage may not be a prerequisite for muscle adaptations and/or that the increased and repeated muscle damage in the alternating group participants prevented them from exerting near maximal effort that is necessary for strength improvements.

## Conflict of Interest

None declared.
